# Characterization of copy numbers of 16S rDNA and 16S rRNA of *Candidatus *Liberibacter asiaticus and the implication in detection *in planta *using quantitative PCR

**DOI:** 10.1186/1756-0500-2-37

**Published:** 2009-03-06

**Authors:** Jeong-soon Kim, Nian Wang

**Affiliations:** 1 Citrus Research and Education Center, University of Florida, 700 Experiment Station Road, Lake Alfred, FL 33850, USA; 2 Department of Microbiology and Cell Science, University of Florida, Gainesville, FL 32611, USA

## Abstract

**Background:**

Citrus Huanglongbing (HLB) is one of the most devastating diseases on citrus and is associated with *Candidatus *Liberibacter spp.. The pathogens are phloem limited and have not been cultured *in vitro*. The current management strategy of HLB is to remove infected citrus trees and reduce psyllid populations with insecticides to prevent the spreading. This strategy requires sensitive and reliable diagnostic methods for early detection.

**Results:**

We investigated the copy numbers of the 16S rDNA and 16S rRNA of the HLB pathogen and the implication of improving the diagnosis of HLB for early detection using Quantitative PCR. We compared the detection of HLB with different Quantitative PCR based methods with primers/probe targeting either 16S rDNA, beta-operon DNA, 16S rRNA, or beta-operon RNA. The 16S rDNA copy number of *Ca*. Liberibacter asiaticus was estimated to be three times of that of the beta-operon region, thus allowing detection of lower titer of *Ca*. L. asiaticus. Quantitative reverse transcriptional PCR (QRT-PCR) indicated that the 16S rRNA averaged 7.83 times more than that of 16S rDNA for the same samples. Dilution analysis also indicates that QRT-PCR targeting 16S rRNA is 10 time more sensitive than QPCR targeting 16S rDNA. Thus QRT-PCR was able to increase the sensitivity of detection by targeting 16S rRNA.

**Conclusion:**

Our result indicates that *Candidatus *Liberibacter asiaticus contains three copies of 16S rDNA. The copy number of 16S rRNA of *Ca*. L. asiaticus *in planta *averaged about 7.8 times of 16S rDNA for the same set of samples tested in this study. Detection sensitivity of HLB could be improved through the following approaches: using 16S rDNA based primers/probe in the QPCR assays; and using QRT-PCR assays targeting 16S rRNA.

## Background

Citrus Huanglongbing (HLB) is one of the most devastating diseases on citrus and is associated with a phloem limited bacterium which has yet to be cultured *in vitro*. Consequently, the pathogen was given a provisional *Candidatus *status in nomenclature [1,2]. Currently, three species of the pathogen are recognized from trees with HLB disease based on 16S rDNA sequence: *Candidatus *Liberibacter asiaticus (Las), *Ca. *Liberibacter africanus (Laf), and *Ca. *Liberibacter americanus (Lam); Las is the most prevalent species among HLB infected trees [1,3-5]. Las has been spreading worldwide over the last century and has been identified in Japan, China, Southeast Asia, India, Arabian Peninsula, Brazil, Florida and other citrus producing areas [3,4]. The current management strategy of HLB is to remove infected citrus trees and reduce psyllid populations with insecticides to prevent it from spreading. This strategy requires sensitive and reliable diagnostic methods for early detection.

The conventional way of diagnosing HLB is based on visual assessment of symptoms. Typical symptoms of HLB of infected trees include blotchy mottle and/or variegated chlorosis of leaves, pale yellow leaves, and stunting. The leaves become upright, followed by leaf drop from the laminar or petiole abscission zones, and at later stages extensive twig dieback occurs [3]. Often small-sized, lop-sided, and bitter tasting fruits with aborted seeds are found on HLB-affected trees. However, these symptoms seem not to be HLB specific since a *Phytoplasma *sp. was reported to cause very similar symptoms in citrus in Brazil [6]. It is also reported that Las can survive for years in citrus before showing obvious symptoms. HLB symptoms also vary with environment and infected trees become less symptomatic under high temperature during the summer.

To overcome the shortcomings of symptom-based diagnosis, various detection methods have been developed in recent years. DNA probes, conventional and Quantitative PCR assays, electron microscope, enzyme-linked immunosorbent assays (ELISA) and biological indexing have been reported to be used for successful diagnosis [3,7,8]. In recent years, diagnosis of HLB based on PCR methodology (Conventional PCR and Quantitative PCR) has gained popularity due to its sensitivity and reliability [7,8]. In this study, we investigated the copy numbers of the 16S rDNA and 16S rRNA of Las and the implication of improving the diagnosis of HLB for early detection using either QPCR or QRT-PCR.

## Methods

### Plant materials and extraction of DNA and RNA

Citrus leaf samples were collected from the HLB symptomatic and asymptomatic sweet orange (*Citrus sinensis*) trees (about 5-year-old) from one citrus grove which has been confirmed to be infected with *Ca*. Liberibacter asiaticus previously in Polk County, Florida, USA. Only leaves with typical blotchy mottle symptoms were used for symptomatic samples. The leaves were washed in tap water and surface sterilized in 35% bleach (2% active Cl^-^) and 70% (v/v) ethanol for 2 min each and rinsed three times with sterile water. Midribs were separated from leaf samples and cut into pieces. 0.1 g of tissue (fresh weight) from each sample was frozen in liquid nitrogen for DNA and RNA extraction, respectively. Midribs were chosen since they are phloem rich as Las is known to be phloem limited. DNA from plant samples was extracted using the Wizard^® ^Genomic DNA purification kit (Promega, Madison, WI, USA) following the protocol for isolating genomic DNA from plant tissue and dissolved in 100 μl of water. RNA was extracted using the RNeasy mini kit (Qiagen, Valencia, CA, USA) following the manufacturers' instructions and dissolved in 100 μl of water.

### Quantitative PCR (QPCR)

All QPCR assays were performed using ABI PRISM 7500 Sequence detection system (Applied Biosystems, Foster City, CA, USA). The Primer/probe set, CQULA04F-CQULAP10-CQULA04R, was used to target the β-operon region of Las. For 16S rDNA, QPCR was carried out with the primers and probe HLBas/HLBr/HLBp for Las essentially as described in Li et al. [7]. Both primer/probe sets have been successfully used for diagnosis and detection. The specificity of primer/probe CQULA04F-CQULAP10-CQULA04R and HLBas/HLBr/HLBp has been confirmed previously [7,8]. The probes were labelled with 56-FAM as a reporter fluorescent dye at the 5' end and with 3BHQ_1 as the quencher dye. QPCR reactions were performed according to the condition described previously with modification [8]. Briefly, QPCR reactions were performed in a 25 μL reaction using 2× Quantitect Probe PCR master mix (Qiagen, Valencia, CA, USA), 0.8 μM of each primer, 0.4 μM of probe (IDT, Coralville, IA, USA), and an appropriate amount of template DNA. The PCR conditions were 50°C for 2 min, 95°C for 15 min, 45 cycles of each 94°C for 15 sec and 60°C for 1 min. Each individual QPCR assay had at least 3 replications. Results were analyzed using ABI Prism software. Raw data were analyzed using the default settings (threshold = 0.2) of the software. DNA samples extracted from healthy citrus were used as negative control.

### Quantitative Reverse Transcriptional PCR (QRT-PCR)

QRT-PCR was used to detect Las 16S rRNA or beta-operon RNA using the QuantiTect Probe RT-PCR Kit (Qiagen) following the manufacturer's instructions. The same primer/probe HLBas/HLBr/HLBp and CQULA04F-CQULAP10-CQULA04R for QPCR targeting 16S rDNA and β-operon region were used for QRT-PCR assays, respectively [7]. Reverse transcription was conducted at 50°C for 30 min with 1 μl of total RNA as template, then followed by initial activation of HotStarTaq DNA Polymerase (95°C, 15 min). Totally, 45 cycles of reactions (94°C for 15 sec, 60°C for 60 sec) were performed. Eight samples were used for QRT-PCR assays and each individual QRT-PCR assay had 3 technical repeats. For dilution study, head to head study was conducted for DNA and RNA extracted from the same set of samples. Both DNA and RNA were diluted from 10 to 10^7 ^times and 1 μl of DNA or RNA was used for each QPCR or QRT-PCR assay, respectively.

## Results and discussion

### Comparing the gene copy number of 16S rDNA and beta-operon

Among the bacterial species, the 16S rDNA copy number varies considerably from 1 to 15 [9,10]. Thus, it is possible to target the high copy number gene for better sensitivity. QPCR has been shown to be able to determine the rDNA copy [11]. In this study, the standard equations, y = -0.3101x + 12.09 and y = -0.288x + 11.61, which were modified from the equations previously developed for 16S rDNA and beta-operon of Las respectively, to fit the conditions used in this study, were used to quantify the Las bacterial population as genome equivalents [7,8,12]. Totally, eight samples were used for calculation. For the same set of samples, the Las population based on the 16S rDNA method averaged 3.15 ± 0.11 (SD) of that calculated by beta-operon method. Thus it is estimated that the copy number of 16S rDNA is three times of beta-operon. Examination of all sequenced bacteria indicated that beta-operon is one copy in bacteria. If this is also true for *Ca*. Liberibacter species, the copy number for Las 16S rDNA should be three copies. By the time this manuscript was accepted, it is also learned that *Ca*. Liberibacter americanus contains three copies of 16S rDNA [N. Wulff pers. comm.]. The draft genome sequence of *Ca*. Liberibacter asiaticus is also in accordance with our result [Y.P. Duan pers. comm.]. Thus compared to QPCR assays targeting beta-operon, a 16S rDNA based QPCR assay is likely to be more sensitive due to its higher copy number per genome.

### Quantitative Reverse Transcriptional PCR (QRT-PCR) targeting 16S rRNA

It has been reported that 16s rRNA could reach up to 10^4^–10^5 ^copies per cell [13]. Thus, 16S rRNA should be a good target to increase the sensitivity of detection. To calculate the copy numbers of 16S rDNA and 16S rRNA, leaf samples from known HLB pathogen infected trees and healthy trees were aliquoted equally for DNA and RNA extraction and dissolved into 100 μl water, respectively. 1 μl of DNA or RNA samples from a total volume of 100 μl were used for either QRT-PCR or QPCR assays, respectively. The same equation was used to calculate the 16S rRNA and 16S rDNA copy number since the same sets of primer/probe HLBas/HLBr/HLBp were used for both QRT-PCR and QPCR assays. The 16S rRNA copy number is 7.83 ± 4.12 (SD) times of that of 16S rDNA for the same samples (Table 1). Similarly, the same equation was used to quantify the DNA and RNA copy numbers of beta-operon region. Dilution analysis of the QRT-PCR targeting 16S rRNA indicates that the RNA sample tested could be detected up to 10^5 ^dilution (Table 2). However, QPCR targeting 16S rDNA could detect the Las up to 10^4 ^dilution for the same set of samples. The dilution analysis for both assays fitted the exponential relationship between Ct value and dilution. Inconsistent results were obtained for further dilution in both assays, thus were considered negative. This result suggests that QRT-PCR assay targeting 16S rRNA is about ten times more sensitive than QPCR assay targeting 16S rDNA. This data is in accordance with the fact that the 16S rRNA copy number is 7.83 ± 4.12 (SD) times of that of 16S rDNA for the same set of samples tested in this study. However, it is surprising that the 16S rRNA is only 7.83 times of that of 16S rDNA as 16s rRNA has been reported to reach up to 10^4^–10^5 ^copies per cell [13]. This is probably due to the slow growth of *Ca*. Liberibacter asiaticus *in planta *[14]. Past studies indicate that bacterial growth rate is highly correlated to various measures of RNA content [15]. This is consistent with the difficulty of detecting *Ca*. Liberibacter asiaticus using Fluorescent in situ hybridization targeting 16S rRNA [N. Wang unpublished]. The high ratio of dead cells/live cell contributes to the unexpected low 16rRNA copy number. Our previous study indicated that viable Las cell rate ranging from 17% to 31% in different citrus samples using QPCR with the aid of ethidium monoazide which can differentiate viable cells from dead cells [13,16-18]. The lower copy number of the beta-operon RNA than the beta-operon DNA also suggests the majority of Las cells *in planta *are dead cells, which consequently contributes to the unexpected low rRNA/rDNA ratio. It is also possible that beta-operon RNA is not stable RNAs (which mainly contain rRNA and tRNA), and subjects to massive loss due to degradation in the sample preparation. Still, QRT-PCR assay targeting 16S rRNA is 10 times more sensitive than QPCR. The challenge for QRT-PCR is handling RNA since it is more sensitive to degradation while DNA is quite stable. Plus, QRT-PCR assay is more expensive and more time-consuming than QPCR assay. Thus, optimization is needed to address those issues in order to make QRT-PCR practical in diagnosis.

**Table 1:** Comparison of copy numbers of 16S rDNA, beta-operon DNA, 16S rRNA, and beta-operon RNA

Copy number	Average	SD
beta-operon DNA	130316.70	17022.84
16S DNA	410145.75	51623.13
beta-operon RNA	1517.15	972.17
16S RNA	3211824.58	1899947.61
16S DNA/beta-operon DNA	3.15	0.11
16S RNA/16S DNA	7.83	4.12
beta-operon DNA/beta-operon RNA	85.90	71.93

Each citrus sample was divided into two Eppendorf tubes with 0.1 g each for DNA or RNA extraction and dissolved into 100 μl of water, respectively. 1 μl of DNA or RNA samples was used in either QPCR or QRT-PCR assays respectively. Totally, eight samples were used for calculation.

**Table 2:** Comparison of QRT-PCR and QPCR assays

Dilution	Ct value	SD	Ct value change	SD
QPCR				
10	23.87	0.02		
10^2^	27.16	0.17	3.29	0.17
10^3^	30.47	0.31	3.30	0.48
10^4^	33.93	0.20	3.46	0.11
QRT-PCR				
10	20.60	0.01		
10^2^	23.77	0.08	3.17	0.08
10^3^	26.82	0.14	3.06	0.18
10^4^	29.53	0.12	2.70	0.12
10^5^	32.99	0.76	3.47	0.80

Each citrus sample was divided into two Eppendorf tubes with 0.1 g each for DNA or RNA extraction and dissolved into 100 μl of water, respectively. 1 μl of DNA or RNA samples was used in either QPCR or QRT-PCR assays respectively. Each value is the mean of three replicates.

Our previous study indicated that a minimum bacterial concentration was required for HLB symptom development in studying the population of Las in symptomatic and asymptomatic leaves [14]. Thus improvement of detection sensitivity of Las could lead to early detection of HLB without relying on symptoms on citrus.

## Conclusion

Our result indicates that Las contains three copies of 16S rDNA. The copy number of 16S rRNA of Las in *planta *averaged about 7.8 times of 16S rDNA. QRT-PCR targeting 16S rRNA was 10 times more sensitive than QPCR targeting 16S rDNA. Detection sensitivity of HLB could be improved through the following approaches: using 16S rDNA based primers/probe in the QPCR assays; and using QRT-PCR assays targeting 16S rRNA.

## Competing interests

The authors declare that they have no competing interests.

## Authors' contributions

J-sK and NW conducted the experiments. NW wrote the manuscript. All authors read and approved the final manuscript.

## References

[B1] Jagoueix S, Bové JM, Garnier M (1994). The phloem-limited bacterium of greening disease of citrus is a member of alpha subdivision of the Proteobacteria. Int J Syst Bacteriol.

[B2] Murray RGE, Schleifer KH (1994). Taxonomic notes: a proposal for recording the properties of putative taxa of procaryotes. Int J Syst Bacteriol.

[B3] Bové JM (2006). Huanglongbing: a destructive, newly-emerging, century-old disease of citrus. J Plant Pathol.

[B4] da Graça JV (1991). Citrus greening disease. Ann Rev Phytopathol.

[B5] Teixeira DC, Ayres J, Danet JL, Jagoueix-Eveillard S, Saillard C, Bové JM (2005). First report of a huanglongbing-like disease of citrus in São Paulo, Brazil, and association of a new Liberibacter species, "*Candidatus *Liberibacter americanus" with the disease. Plant Dis.

[B6] Teixeira DC, Wulff NA, Martins EC, Kitajima EW, Bassanezi R, Ayres AJ, Eveillard S, Saillard C, Bové JM (2008). A phytoplasma closely related to the Pigeon Pea Witches'-Broom Phytoplasma (16Sr IX) is associated with citrus huanglongbing symptoms in the state of São Paulo, Brazil. Phytopathology.

[B7] Li W, Hartung JS, Levy L (2006). Quantitative real-time PCR for detection and identification of *Candidatus *Liberibacter species associated with citrus huanglongbing. J Microbiol Meth.

[B8] Wang Z, Yin Y, Hu H, Yuan Q, Peng G, Xia Y (2006). Development and application of molecular-based diagnosis for '*Candidatus *Liberibacter asiaticus', the causal pathogen of citrus huanglongbing. Plant Pathol.

[B9] Klappenbach JA, Dunbar JM, Schmidt TS (2000). rRNA operon copy number reflects ecological strategies of bacteria. Appl Environ Microbiol.

[B10] Charles H, Ishikawa H (1999). Physical and genetic map of the genome of *Buchnera*, the primary endosymbiont of the pea aphid *Acyrthosiphon pisun*. J Mol Evol.

[B11] Lee C, Kim J, Shin SG, Hwang S (2006). Absolute and relative QPCR quantification of plasmid copy number in *Escherichia coli*. J Biotechnol.

[B12] Tatineni S, Sagaram US, Gowda S, Robertson CJ, Dawson WO, Iwanami T, Wang N (2008). In plant distribution of '*Candidatus *Liberibacter asiaticus' as revealed by polymerase chain reaction (PCR) and real-time PCR. Phytopathology.

[B13] Zwirglmaier K, Ludwig W, Schleifer KH (2004). Recognition of individual genes in a single bacterial cell by fluorescence in situ hybridization – RING-FISH. Mol Microbiol.

[B14] Trividi P, Sagaram US, Brlansky RH, Rogers M, Stelinski LL, Oswalt C, Kim JS, Wang N (2009). Quantification of viable *Candidatus *Liberibacter asiaticus in hosts using Quantitative PCR with the aid of ethidium monoazide (EMA). Eur J Plant Path.

[B15] Kemp PF, Lee S, LaRoche J (1993). Estimation of the growth rate of slowly growing marine bacteria from RNA content. Appl Environ Microbiol.

[B16] Nocker A, Camper AK (2006). Selective removal of DNA from dead cells of mixed bacterial communities by use of ethidium monoazide. Appl Environ Microbiol.

[B17] Rudi K, Moen B, Drømtrop SM, Holck AL (2005). Use of ethidium monoazide and PCR in combination for quantification of viable and dead cells in complex samples. Appl Environ Microbiol.

[B18] Wang S, Levin RE (2006). Discrimination of viable *Vibrio vulnificus *cells from dead cells in real-time PCR. J Microbiol Methods.

